# Single-copy Snail upregulation causes partial epithelial-mesenchymal transition in colon cancer cells

**DOI:** 10.1186/s12885-023-10581-3

**Published:** 2023-02-14

**Authors:** Fatima Junaid, Goran Tomic, Richard Kemp, Doug J. Winton

**Affiliations:** Cancer Research UK – Cambridge Institute, University of Cambridge, Cambridge, CB2 0RE UK

**Keywords:** Epithelial-mesenchymal transition, SNAIL, Partial transition, Metastasis

## Abstract

**Background:**

Epithelial-mesenchymal transition (EMT) is an embryonic programme implicated in cancer stem cells, metastasis and therapeutic resistance. Its role in cancer progression remains controversial because the transition can be partial or complete in different models and contexts.

**Methods:**

Using human colon cancer DLD-1 cells, we engineered a cell line with a single-copy of Snail that was doxycycline-inducible and compared it to existing EMT models in DLD-1. The effect of Snail upregulation was characterised functionally, morphologically, and by transcriptional profiling and protein expression.

**Results:**

Induction with doxycycline increased Snail expression to a level similar to that observed in cancer cell lines spontaneously expressing Snail and results in partial EMT. In comparison, higher levels of overexpression arising from introduction of episomal-Snail, results in complete EMT. DLD-1 cells with partial EMT show chemoresistance in vitro, increased tumour growth in vivo and decreased apoptosis.

**Conclusions:**

These findings highlight that the amount of bioavailable Snail can dictate phenotypic outcome and that partial EMT may be a preferred outcome of models operating within a natural range of Snail overexpression.

**Supplementary Information:**

The online version contains supplementary material available at 10.1186/s12885-023-10581-3.

## Background

Epithelial to mesenchymal transition (EMT) is an embryological programme during which cells lose epithelial characteristics of cell polarity, stability and cell–cell adhesion and acquire mesenchymal markers, morphology and motility [1, 2]. EMT is implicated in cancer initiation, invasion and metastasis but its role in cancer is controversial.

Upregulation of genes mediating EMT, such as Slug or Snail in breast or colon cancers and Twist1 in melanoma, lead to increased invasive characteristics and increased tumorigenicity in vitro and in vivo [3–5]*.* However, the necessity of EMT in metastasis of epithelial cancers has been questioned by in vitro evidence that kidney and breast cancer epithelial cells can move by collective migration [6, 7]. Seminal experiments using mouse models of breast and pancreatic cancer further claimed no EMT was required in metastases, but only that EMT leads to chemoresistance and post-therapeutic tumour recurrence [8, 9]. This interpretation has been challenged on the basis that EMT can be variable in extent in different cancer subtypes and its recognition depends on markers of mesenchymal cells and therefore only complete EMT has been evaluated [10].

A more recent concept has been the identification of an intermediate state, or partial EMT (pEMT), where cells express both epithelial and mesenchymal markers or characteristics. Evidence for a metastable, pEMT has been found in the contexts of renal and cochlear development and kidney fibrosis [11–14]. Partial EMT has also been identified and associated with the poorest survival in breast cancer patients [14], as well as theoretically deduced in mathematical models of cancer development [15]. Studies in pancreatic and breast cancer found that only cells that had undergone partial not complete EMT could lead to metastases, and those in a more intermediate state were more likely to metastasise to the liver than the lung and were associated with poorer prognosis [16–18]. High Snail expression was found to cause pEMT in basal breast cancer cells and was essential for tumorigencity, but Zeb1 was required for complete EMT [19]. A lineage tracing mouse model of pancreatic cancer was used to suggest that pEMT is due to relocalisation of epithelial proteins, whereas complete EMT is due to transcriptional repression of epithelial proteins [20].

A number of studies have also identified pEMT in colorectal cancer models. Partial EMT was induced in Drosophilia intestinal tumours by overexpression of Snail, leading to collective migration and metastases [21]. Incubating colorectal cancer cell line DLD-1 with both pro-mesenchymal TGF-β and pro-epithelial VEGF-A for 48 h, caused increased epithelial E-cadherin, mesenchymal vimentin and pro-EMT transcription factor Slug [22]. This led to increased surface migration and invasion. A cell line developed from human colon cancer circulating tumour cells was also found to express both Snail and E-cadherin [23].

This body of work indicates a process that is context- and cancer-dependent, a corollary of which must be that different models will similarly show different phenotypic outcomes depending on how EMT is mediated and measured [10, 24–27]. With such inconsistency between EMT models and a lack of validation of how closely they recapitulate in vivo processes, a robust, reproducible, more-physiological model is required, to enable further analysis of the effect of EMT on cancer.

Here, to explore the effect of different amounts of bioavailable Snail we developed an inducible model of EMT in the colon cancer cell line DLD-1, by single-copy insertion of the Snail gene in a defined genomic region. DLD-1 was selected as a cell line with mutations typical of colorectal cancer, including a truncated Adenomatous polyposis coli (APC) protein and heterozygous dominant negative mutations in KRAS and TP53, but without raised Snail expression [28, 29]. We found that induction of physiological levels of Snail led to a partial EMT phenotype in vitro and in vivo.

## Methods

### Cell culture

Cells were cultured in Dulbecco’s Modified Eagle Medium (Gibco) containing 4.5 g/L D-Glucose and L-Glutamine and supplemented with 10% tetracycline-free foetal bovine serum (Clontech) and 1% penicillin and streptomycin (Gibco). Cells were passaged when confluent with 0.05% trypsin (Gibco) and incubated in a Heracell 150 incubator, 20% oxygen and 5% carbon dioxide at 37 °C. DLD-1 cells were a kind gift from Stephen Taylor, University of Manchester. HCT-116, SW480, and SW620 cells were from group stocks. All cell lines were verified by short-tandem repeat genotyping undertaken by the in-house Biorepository. Cell cultures were regularly tested for mycoplasma. Antibiotics were added for selection in the following concentrations: Blasticidin S HCl (Invitrogen) 8 μg/ml, hygromycin B (Invitrogen) 400 μg/ml, puromycin (Invitrogen) 3.5 μg/ml, and zeocin (Sigma) 60 μg/ml.

### Cloning

Snail^wt^ and Snail^lo^: DNA fragments were isolated from restriction digest or polymerase chain reaction of plasmids, assembled by ligation using Infusion Cloning (Clontech) and transformed into XL-2 Ultracompetent Blue Cells (Agilent Technologies) by heatshock. Plasmids were extracted from bacteria, sequence verified by Sanger sequencing, then inserted into a DLD-1 Flp-In T-Rex cell line containing a single Frt site (a kind gift from Professor Steve Taylor, University of Manchester) following the protocol in Tomic et al., 2018 [30]. The copy number and location of integration of plasmids into cells were verified by loss of b-galactosidase staining, PCR and quantitative PCR, protocol as in Tomic et al., 2018. Details of primers and plasmid construction can be found in Sup. Table 2 and single copy confirmation of Snail in Sup. Fig. 2. The location of the frt site in DLD-1 Flp-In T-Rex cells was identified using the Universal GenomeWaler 2.0 Kit (Clontech) and following standard protocols. Snail^hi^: protocol followed from Siemens et al., 2011.

### Gene expression

Gene expression was measured by qPCR. The following Taqman probes were used: E-cadherin (Hs01023894 m1), N-cadherin (Hs00983056 m1), RPL19 (Hs02338565 gH), Snail (Hs00195591 m1) and vimentin (Hs00185584 m1) (all Thermo Fisher Scientific). Technical and biological repeats were both run in triplicate. Plates were run on a QuantStudio 6 Flex Real-Time PCR System (Thermo Fisher Scientific) with the following programme: 95 °C 20 s then 40 cycles of 95 °C 3 s and 60 °C 30 s. Data was analysed using the Ct method normalised to RPL19.

A multiplex qPCR of 84 EMT-associated genes was conducted using the RT^2^ Profiler PCR Array Human Epithelial to Mesenchymal Transition (QIAGEN) according to the manufacturer’s instructions.

### Scratch wound assay

Cells that had been incubated with doxycycline for 14 days were plated at 90% confluence in Essen Image-Lock 24-well plates. A 1 mm scratch was drawn across the centre of the well using 10ul pipette tips and a wound maker. Plates were loaded into an IncuCyte ZOOM System inside a 37 °C incubator. Images of the wound were taken every 3 h and measurements calculated by the system’s software.

### Transwell migration assay

Cells were cultured in serum levels stated for each experiment for 24 h then 2 × 10^5^ were seeded in triplicate into the top chamber of 6.5 mm Corning Transwells with 8 μM pores. Transwells were placed in 24-well plates filled with stated serum levels in each experiment and incubated at 37 °C for 24 h. Media was aspirated from the plate and the upper membrane wiped with water and cotton to remove cells that had not migrated. Transwells were fixed in methanol (Sigma-Aldrich) for 10 min, incubated in Giemsa Stain (Fluka Analytical) for 10 min, washed several times in water then left to dry at 25 °C for 24 h. The membrane was excised and fixed between a slide and a cover slip. The number of visible nuclei in three fields at a 40 × objective on a Nikon Eclipse 50i microscope was counted and averaged.

### Immunofluoresence

Single cell suspensions (4 × 10^4^) were seeded onto 8-well μ-slides (ibidi) and fixed with 4% paraformaldehyde at room temperature for 15 min. Antibodies used were as follows: E-cadherin (BD Transduction Laboratories, 1:100) with AlexaFluor 647 monkey anti-mouse IgG (Invitrogen 1:500); Snail (Santa Cruz SC-10433, 1:50) with AlexaFluor 555 donkey anti-goat 1gG (Invitrogen, 1:500); Vimentin (Sigma C9050, 1:400), and DAPI (Invitrogen, final concentration 300 nM).

### Western blot

Cells were lysed with Laemmli buffer for protein collection then 33 μg total protein was loaded into each well of a 4–12% Bis–Tris gel (Thermo Fisher Scientific). Antibodies used were as follows: E-cadherin (Abcam ab1416, 1:1000) with IRDye 800CW goat anti-mouse (Li-Cor Biosciences, 1:5000); GAPDH (Sigma G8795, 1:1000) with IRDye 800CW goat anti-mouse (Li-Cor Biosciences, 1:5000); N-cadherin (Cell Signaling D4R1H, 1:1000) with IRDye 680LT goat anti-rabbit (Li-Cor Biosciences, 1:20,000); Snail (Cell Signaling C15D3, 1:1000) with IRDye 680LT goat anti-rabbit (Li-Cor Biosciences, 1:20,000); and Vimentin (Cell Signaling D21H3, 1:1000) with IRDye 680LT goat anti-rabbit (Li-Cor Biosciences, 1:20,000).

### Histology

Xenografts were incubated in 4% paraformaldehyde for 24 h at 4 °C then remained in 70% ethanol until processed for paraffin wax embedding by the in-house histology core facility. Antibodies used were as follows: β-catenin (BD Biosciences 610,154, 1:100); cleaved caspase-3 (Cell Signaling Technology 9664, 1:200); E-cadherin (Daki M3612, 1:25); human Ki67 (Dako M7240, 1:400); Vimentin (Novocastra NCL-L-VIM-572, 1:400); and secondary antibodies from the Bond Polymer Refine Kit.

### Chemoresistance assay

Cells were maintained in DOX or dimethyl sulfoxide (DMSO) for 14 days then 3 × 10^4^ cells per well were seeded onto 24-well plates and loaded into an IncuCyte ZOOM System inside a 37 °C incubator, and maintained in DOX or DMSO. After 24 h, the media was removed and replaced with oxaliplatin or 5-fluoruracil, still in DOX- or DMSO-rich media. Images of the wells were taken every 3 h and the confluence measured at each time point as calculated by the system’s software.

### Imaging

Phase contrast images of live cells were taken on a Zeiss Axiovert Microscope. Images of tumour histology were taken on a Nikon Eclipse 50i microscope. Immunofluorescent images of cells were taken on a Leica Tandem Confocal microscope using LAS-AF software.

### Animal work

All mouse work was conducted in line with UK Home Office regulations. Mice were kept on a 12-h light–dark cycle in individually ventilated cages with free access to food and drink. Standard diet (5R58, LabDiet) or doxycycline supplemented diet (200 ppm, 5058, LabDiet) was provided, as per requirements of the experiment. Mice were checked once per day by staff for signs of disease progression affecting eating, breathing or locomotion. All mice used were non-obese diabetic severe combined immune deficiency interleukin 2 receptor-null (NSG) bred by Alexandra Bruna (in-house, CRUK-CI). No animals were excluded from the final analysis.

### Subcutaneous injections

Cells were counted on a Vi-Cell counter (Beckmann Coulter) and resuspended in 1:1 growth factor reduced matrigel (Corning) and cell culture media at a concentration of 10^5^/ml. Under anaesthetic, mice were injected with 100 μl cell suspension in each flank. Mice were alternately allocated to each group, ensuring equal numbers of mice in each group in each cage. Twice weekly measurements were taken of total tumour mass with electronic scales and tumour size with digital callipers by a single individual blinded to group allocation.

### Statistical analysis

Significance values for Student’s t-test, analysis of variance (ANOVA) and Kruskal–Wallis test were calculated on statistical package GraphPad Prism. Comparison of the means between multiple samples was then conducted by Tukey’s multiple comparisons test for ANOVA and Dunn’s test for Kruskal- Wallis tests. Significance of non-linear fit, comparing the differences in gap closure in a scratch wound assay, was calculated on GraphPad Prism. Statistical analysis of the multiplex PCR was calculated in the PCR Array Data Analysis Software (QIAGEN). Significance of the rates of tumour growth were based on the linear regression of interaction between conditions calculated using the programme R. This compares the likelihood that two data sets follow the same line of best fit.

## Results

### Physiological levels of Snail in CRC cells

To produce a model of EMT that is mediated by levels of Snail overexpression comparable to those that arise spontaneously in cancer cell lines, a single copy expression cassette of human SNAI gene (Snail) that was doxycycline-inducible was engineered into a defined, open, genomic region in DLD-1 cells using frt recombination (Sup. Fig. 1) [31]. This region was identified by PCR-based genome walking as the final intron of *ARID5B* on chromosome 10. Targeted single-copy insertion of Snail and vector-only cassettes was confirmed by PCR, qPCR copy number assay, and ablation of lacZ staining (Sup. Fig. 2). For comparison, Snail was also introduced into DLD-1 cells using a pRTR episome that has been previously published, though here the human coding sequence was substituted for the murine one (Sup. Fig. 1) [26].

**Fig. 1:**
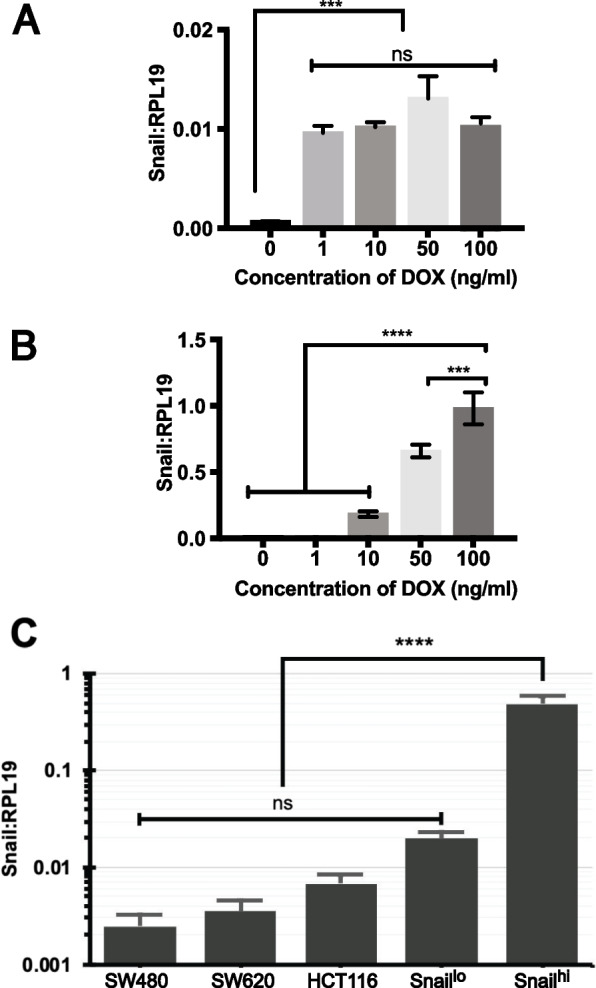
Snail RNA expression with different concentrations of DOX. **A** Snail^lo^ and (**B**) Snail^hi^ (**C**) comparison with CRC cell lines that naturally express Snail. Error bars = SEM. *n* = 3 biological replicates with 3 technical replicates each. Significance based on one-way ANOVA. ns = not significant, ***p < 0.001, ****p < 0.0001

The level of Snail RNA expression was consistent in the single copy Snail line, with an all-or-nothing response to escalating levels of doxycycline (DOX) induction contrasting to dose-dependent responses in episomal-induced Snail cells (Figs. 1a, b). Snail RNA remained near absent expression in the vector-only line (Snail^wt^). When single or episomal Snail cells were cultured with 100 ng/mL DOX, the level of Snail RNA expression in the single-copy Snail line (Snail^lo^) was comparable to other Snail-expressing colon cancer cell lines, whereas the episomal–Snail line (Snail^hi^) expression was 75-fold higher (Fig. 1c).

Snail expression and changes in the transcriptional abundance of responsive genes (E-cadherin and N-cadherin) in Snail^lo^ cells remained similar from 5 to 14 days of DOX induction indicating a stable maintenance of phenotype over this time (Sup. Fig. 3). Subsequently, Snail^lo^ and Snail^wt^ lines were cultured for 14 days in DOX for use in functional assays, using dimethyl sulfoxide (DMSO) as a solvent. Of note Snail^lo^ and Snail^wt^ lines could be cultured in DOX for weeks without an effect on survival (data not shown). In contrast, Snail^hi^ lines could only be cultured for 5 days before mass cell death commenced. (Previous published work with Snail^hi^ used 100 ng/mL DOX and data obtained after 4 to 5 days [14]). To enable comparison of models all cells were incubated in 100 ng/mL DOX but analysed at 4 days for Snail^hi^ and 14 days for Snail^lo^ and Snail^wt^.

### Phenotypic changes in CRC cells based on variable Snail expression

**Fig. 2:**
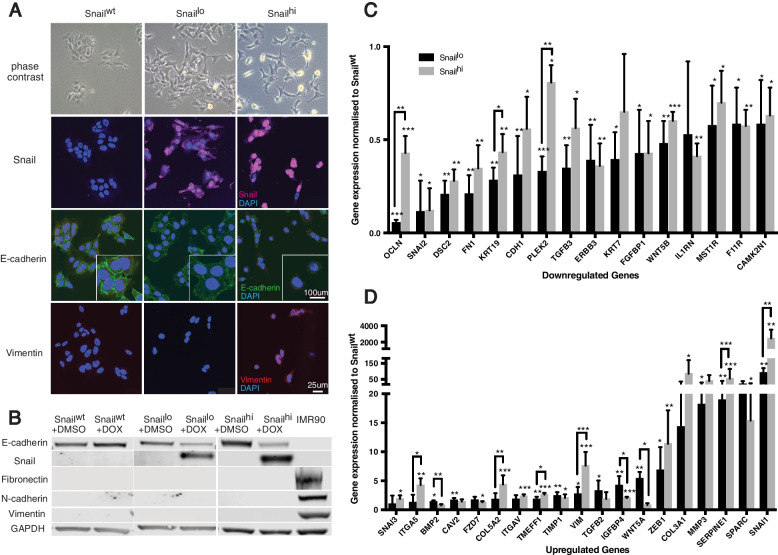
Changes in morphology and marker expression in DLD-1 cells following Snail induction. **A** Phase contrast and immunofluorescence images of cell lines with 100 ng/mL DOX for 14 days (Snail^wt^ and Snail^lo^) or 4 days (Snail^hi^) show acquisition of mesenchymal morphology on Snail expression. **B** Western Blots detecting Snail and downregulation of E- cadherin. IMR90, a fibroblast line, is used as a positive control for mesenchymal proteins (Snail^hi^ and IMR90 from a separate blot). **C**, **D** mRNA array profiling of 84 EMT regulated genes. Snail-expression leads to downregulation (**C**) and upregulation (**D**) of genes, normalised to the Snail^wt^. All comparisons in the presence of DOX. Error bars = 95% confidence interval. *n* = 3. Asterisks over data bars indicate significance from Snail^wt^. Asterisks over connecting lines indicate significance between Snail^lo^ and Snail.^hi^. Student’s t-test. *p < 0.05, **p < 0.01, ***p < 0.001

DLD-1 cells grow with a distinctive cobblestone appearance typical of epithelial cells. Induction of Snail expression resulted in cells being less rounded and more mesenchymal in appearance and with membrane protrusions. These morphological changes towards a mesenchymal phenotype were more pronounced in Snail^hi^ compared to Snail^lo^ expressing cultures (Fig. 2a).

Snail^hi^ lines were confirmed to express more Snail protein compared to Snail^lo^, as shown by immunofluorescence and Western Blot (Figs. 2a, b). Both lines showed a decrease in epithelial E-cadherin protein. On a population basis this decrease was greater in Snail^lo^ than Snail^hi^ cells as shown by Western blotting but E-cadherin was completely absent only in a proportion of Snail^hi^ cells as shown by immunofluorescence. This is likely explained by the different incubation times: 4 days for Snail^hi^ and 14 for Snail^lo^. There were no changes at the protein level of mesenchymal markers N-cadherin or fibronectin in either line. Snail^hi^ cells showed increased vimentin expression by immunofluorescence.

A commercially available mRNA array profiler was used to assess the expression of 84 genes associated with EMT (see Methods). There were widespread decreases of epithelial markers and increases in mesenchymal markers in both cell lines on DOX induction of Snail (Figs. 2c, d, full results Sup. Table 1). Of 84 genes known to be associated with changes in morphology, motility and stemness, the expression of 35 were significantly different between Snail-expressing lines compared to Snail^wt^ controls. In both Snail^lo^ and Snail^hi^ lines there tended to be perturbation of expression in the same genes and with the same trend for up and downregulation. This suggests that the same EMT programme is being exploited. Thus Snail-expression led to a downregulation of genes that encode proteins in cellular adhesion junctions (OCLN, DSC2, CDH1), the epithelial cytoskeleton and its interactors (KRT7, KRT19, PLEK2), cell proliferation (ERBB3, WNT5B) and immunity (IL1RN). There was an upregulation of EMT-TFs (ZEB1, SNAI1) and genes that encode proteins with effects on invasion (ITGAV), mesenchymal cytoskeleton (VIM), cell polarity (WNT5A) and rearrangement of the surrounding matrix (COL3A1, MMP3, SERPINE1). However, a striking difference between the two lines was that decreases in some epithelial markers (OCLN, KRT19, PLEK2) were stronger in Snail^lo^ and increases in some mesenchymal markers (ITGA5, COL5A2, TIMEFF1, VIM, SERPINE1, SNAI1) were greater in Snail^hi^ with the latter being particularly pronounced.

Overall EMT-related gene expression indicate that Snail^lo^ is at least equally efficacious as Snail^hi^ in downregulating epithelial differentiation but that driving a full subsequent mesenchymal differentiation requires the higher levels of Snail expression associated with Snail^hi^.

### Functional effects of partial EMT

The functional effect of Snail upregulation on Snail^lo^ cells was measured by migration and chemoresistance. Snail^hi^ cells died by day 5 of DOX incubation. As DOX treatment itself is well tolerated by Snail^wt^ and Snail^lo^ cells this can be ascribed to high levels of Snail expression being lethal.

**Fig. 3:**
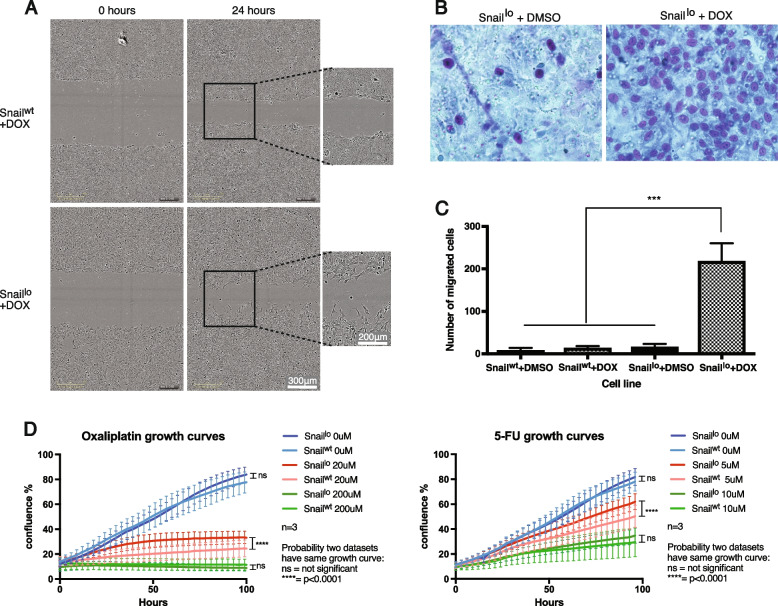
Snail^lo^ cells are motile and chemoresistant. **A** In a scratch wound assay, individual Snail^lo^ cells break away from the leading edge (arrows) unlike Snail^wt^ cell. **B**, **C** Snail^lo^ cells show increased migration in a Transwell migration assay. Light microscopy picture of migrated cells stained with Giemsa stain 24 h after seeding in a 1%:10% serum gradient, (**B**). Quantification of the number of cells that had moved across the membrane (**C**). Error bars = SEM. -/ + DOX. *n* = 3. Significance based on one-way ANOVA, ***p < 0.001. (**D**, **E**) Growth curves in cells incubated with chemotherapeutic agents. Oxaliplatin (**D**) and 5-fluorouracil (5-FU) (**E**). *n* = 3, ****p < 0.0001, ns = not significant

Time-lapse footage in a scratch-wound assay showed Snail^wt^ cells moved collectively, as a sheet, with a straight moving edge, whereas Snail^lo^ cells displayed individual cell migration (Fig. 3a). The moving edge was less uniform and more jagged, with cells displaying mesenchymal-like membrane protrusions. Difference in migratory capacity was confirmed in a Transwell assay. Significantly more Snail^lo^ cells moved across an 8 µm-pore-lined membrane in the presence of DOX than without (Fig. 3b, c). This difference was consistent, regardless of serum gradient across the membrane, discounting a chemotactic effect arising with the experimental design (Sup. Fig. 4).

Snail^lo^ cells showed increased chemoresistance, with higher levels of growth in the presence of oxaliplatin and of 5-fluorouracil, chemotherapeutic agents usually used to treat colorectal cancer (Fig. 3d). This difference in chemoresistance was only evident in moderate drug levels; very high or very low doses showed no difference in growth when compared to controls.

### Partial EMT in *in vivo* tumour growth

To assess the effect of single-copy Snail upregulation in vivo, Snail^lo^ and Snail^wt^ cells were subcutaneously injected into the posterior flanks of NSG mice. All cells were injected in the epithelial, uninduced state, then all mice were started on DOX-chow 7 days later. This was to ensure differences in growth rate were not due to seeding or initial cell survival. All injections produced a tumour (96/96).

**Fig. 4:**
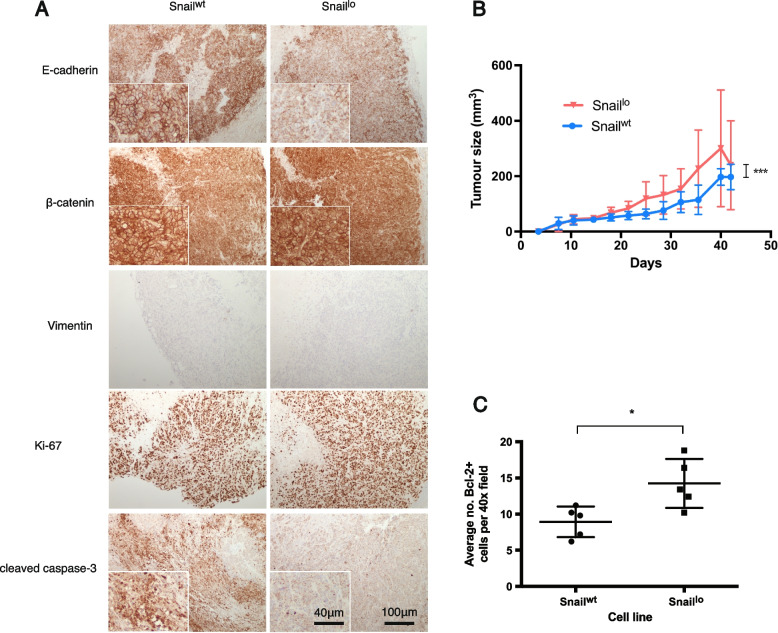
Snail causes a partial EMT in vivo and is anti-apoptotic. Xenografts were harvested from mice 21 days post subcutaneous injection (and 14 days post-DOX induction). **A** Representative images of paraffin sections stained for protein shown by immunohistochemistry. *n* = 4 per condition. **B** Increased growth rate of Snail.^lo^ tumours. There are 30 tumours per condition at day 0 and mice were sacrificed weekly from day 14, removing 6 tumours from each cohort each time. Therefore, *n* = 30 at day 0 and *n* = 6 at day 42. Significance values based on linear regression of interaction between conditions, ***p < 0.001. The number of Bcl-2 + cells in sections of xenograft was counted (**C**). *n* = 5 biological replicates with 5 technical replicates each. Significance based on Student’s t-test, *p < 0.05

Snail^lo^ tumours displayed a partial EMT in vivo, with reduced membranous E-cadherin but no increased vimentin on histology (Fig. 4a). This effect seemed independent of ß-catenin, which remained membrane-bound.

Assessment of tumour size, measured using callipers, showed that Snail^lo^ tumours were significantly larger and grew at a faster rate, when compared to Snail^wt^ tumours (Fig. 4b). This difference in tumour size could not be attributed to proliferation, as both sets of tumours had equivalent levels of Ki-67 staining (Fig. 4a). However, Snail^lo^ tumours showed reduced cleaved caspase-3 staining, a marker for apoptosis, and significantly increased expression of the anti-apoptotic protein Bcl-2 (Fig. 4c). This suggests Snail^lo^ tumours grow faster and larger due to anti-apoptotic mechanisms, rather than increased cell proliferation.

## Discussion

Modest upregulation of the EMT inducer Snail in DLD-1 colon cancer cells leads to a hybrid phenotypic state. This was characterized by downregulation of epithelial markers like E-cadherin, moderate upregulation of mesenchymal proteins, increased motility, and increased chemoresistance. The finding of therapeutic resistance in pEMT cells has also been described previously. HT-29 CRC cells have been shown to have intermediate radioresistance [32]. Surviving cells showed limited downregulation of E-cadherin, moderate increase in vimentin and were highly invasive—characteristics similar to induced Snail^lo^ lines. Wnt3 expression in HER2 breast cancer cells leads to pEMT and decreased sensitivity to trastuzumab [33]. The anti-apoptotic effects observed here are in line with similar effects identified in EMT induction of MDCK cells, immortalized murine mammary cells, and murine hepatocytes [34–36].

By upregulating Snail to different degrees, we were able to show that the extent of EMT is dependent on the level of Snail alone. Moderate overexpression leads to pEMT, significant overexpression leads to complete EMT. Recently it has been suggested that pEMT is a consequence of an alternative pathway affecting protein internalization while the classic transcriptional repression pathways mediated by Snail and others mediate complete EMT [20]. While we have not formally considered the former here, it is clear that in DLD-1 cells, Snail alone is sufficient to induce both partial EMT and complete EMT. Further additional mediators such as Zeb1 are not required in DLD-1 cells to complete the transition, as has been described in basal breast cancer [19].

An area of discussion in pEMT has been the level of stability the intermediate phenotype has. Mathematical modelling was used to show that Snail can induce pEMT in a transient, reversible fashion, while Zeb1 induces complete EMT irreversibly [37]. Another study conducting single cell transcriptomic analysis in head and neck cancer suggested pEMT is transient since pEMT-hi and pEMT-lo cells became indistinguishable after four days in culture [38]. However, the Snail^lo^ model employed here shows that stable, long-lasting pEMT can be imposed by overexpression of Snail alone. Recent studies using lineage tracing in mouse models of skin and breast cancer also provide in vivo evidence of the existence of multiple stable EMT transition states and suggest a continuum of EMT with subpopulations that have distinct functional properties in cancer development [18, 39]. Further modelling suggests that phenotypic stability factors may be responsible for maintaining an intermediate epithelial/mesenchymal phenotype so that it need not be a transient state [40].

It has been shown that prolonged TGF-β exposure in breast cancer cells causes only a partially reversible EMT, leading to increased stem cell expression and chemotherapy resistance when compared with transient TGF-β exposure [41]. It would be interesting to explore whether a similar exposure-dependent effect is observed in the Snail^lo^ model.

## Conclusions

This study highlights that the amount of bioavailable Snail alone can dictate phenotypic outcome and that partial EMT may be a preferred outcome of models operating within a natural range of Snail overexpression.

In vitro models of EMT have been criticized in part because they result in complete EMT while in vivo the extent of the transition may vary. However, such exaggerated models were developed based on the assumption that a complete EMT was the norm in both development and during cancer progression. Refined and simple in vitro models may help determine the extent to which gene dosage and cellular context as opposed to a multiplicity of interacting mediators dictate EMT-related phenotypes. Further research should explore the molecular mechanisms behind differences in phenotypic outcomes between the Snail^hi^ and Snail^lo^ models, and whether the recruitment of other transcription factors, like Zeb1 in breast cancer [19], are involved. 

## Supplementary Information

**Additional file 1.**

**Additional file 2.**

**Additional file 3.**

**Additional file 4.**

**Additional file 5.**

**Additional file 6.**

## Data Availability

The datasets used and/or analysed during the current study are available in the GEO repository, accession number GSE212490.

## Abbreviations

DMSO: Dimethyl sulfoxide
DOX: Doxycycline
EMT: Epithelial-mesenchymal transition
NSG: Non-obese diabetic severe combined immune deficiency interleukin 2 receptor-null
pEMT: Partial epithelial-mesenchymal transition
