# Supplementary material for: Single-copy Snail upregulation causes partial epithelial-mesenchymal transition in colon cancer cells
Source: BMC Cancer. 2023 Feb 14;23:153. doi: 10.1186/s12885-023-10581-3 (PMC9926732; doi:10.1186/s12885-023-10581-3)
Supplement: Supplementary file 1 — Additional file 1. [file 12885_2023_10581_MOESM1_ESM.pdf]

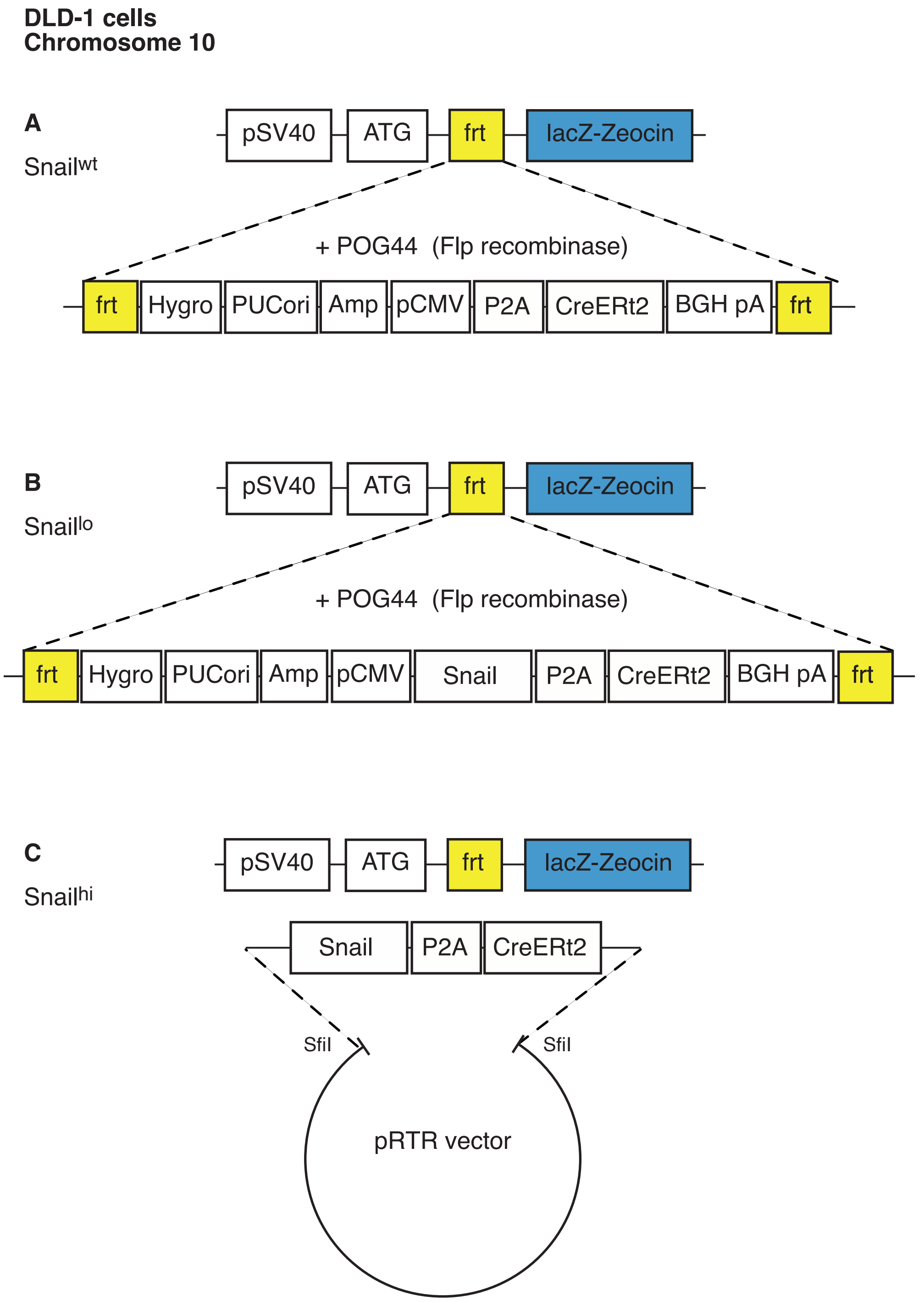

Supplementary Figure 1 - Constructs used to generate inducible EMT cell lines.

Schematic of plasmids used to generate (A) Snail-wt and (B) Snail-lo cells introduced by frt recombination into the previously engineered frt site on chromosome 10 (Holland et al., 2012). (C) Schematic of episome used to generate Snail-hi cells (Siemens et al., 2011).
