# Supplementary material for: Single-copy Snail upregulation causes partial epithelial-mesenchymal transition in colon cancer cells
Source: BMC Cancer. 2023 Feb 14;23:153. doi: 10.1186/s12885-023-10581-3 (PMC9926732; doi:10.1186/s12885-023-10581-3)
Supplement: Supplementary file 2 — Additional file 2. [file 12885_2023_10581_MOESM2_ESM.pdf]

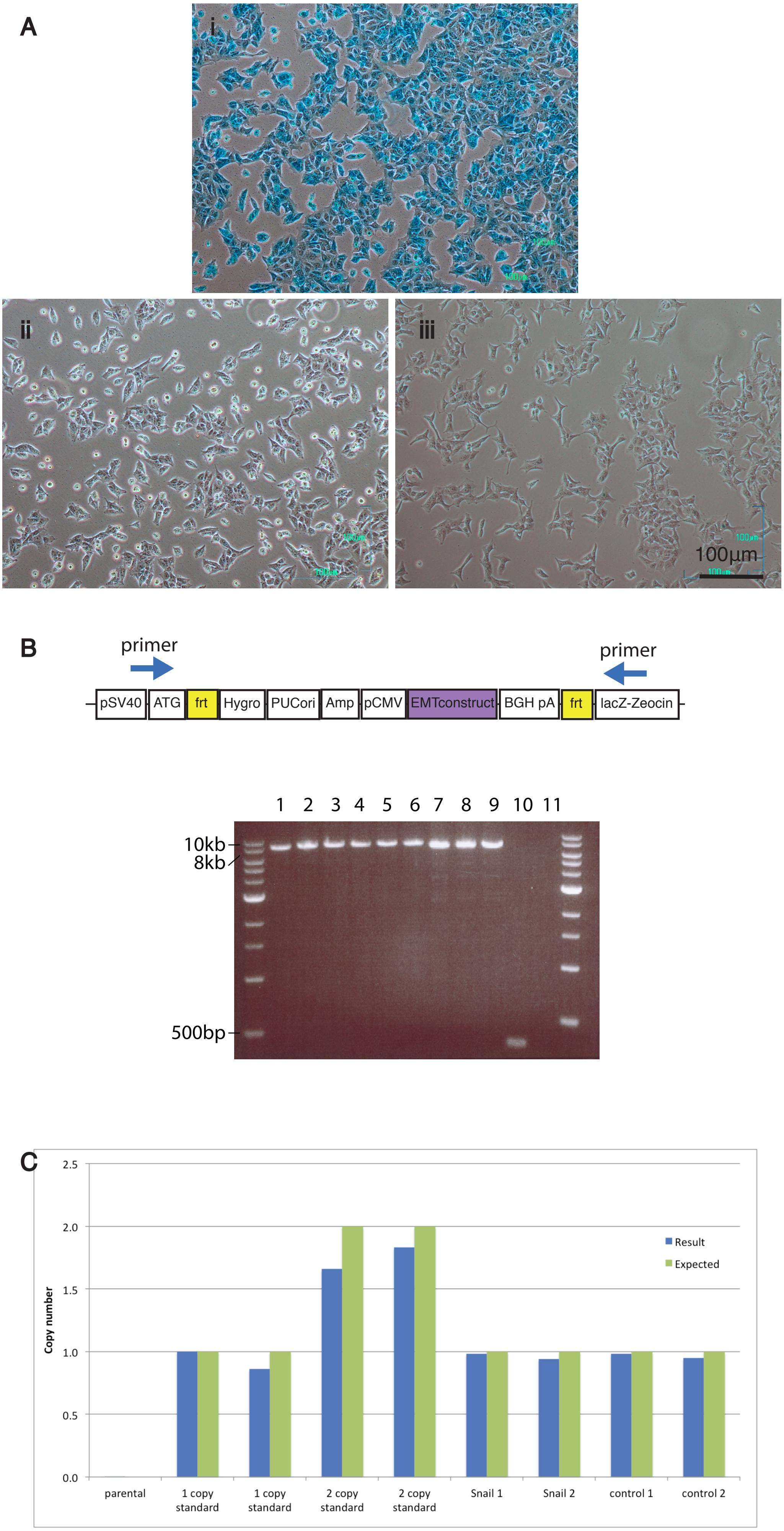

Supplementary Figure 2 - Single copy insertion of cloned Snail-wt and Snail-lo lines

(A) Insertion of the construct (Snail-wt or Snail-lo) was identified initially by loss of lacZ staining (i. untransfected, ii. Snail-wt, iii. Snail-lo). (B) PCR confirmation of insert using primers either side of the FRT site. 10kb ladder. Expected size of PCR product in brackets. Lanes 1-6: Snail-lo (8.3kb); 7-9: Snail-wt (7.6kb); 10: parental (347bp); 11: DNA-free control. (C) qPCR copy number assay using a hygromycin probe normalised RNaseP. There were 10 Snail-wt and 17 Snail-lo non-sister clones, each the derivative of a different single cell. P = parental line.
