# Supplementary material for: Single-copy Snail upregulation causes partial epithelial-mesenchymal transition in colon cancer cells
Source: BMC Cancer. 2023 Feb 14;23:153. doi: 10.1186/s12885-023-10581-3 (PMC9926732; doi:10.1186/s12885-023-10581-3)
Supplement: Supplementary file 3 — Additional file 3. [file 12885_2023_10581_MOESM3_ESM.pdf]

qPCR Snail^{lo} 5d vs 14d
***p<0.001
***p<0.0001

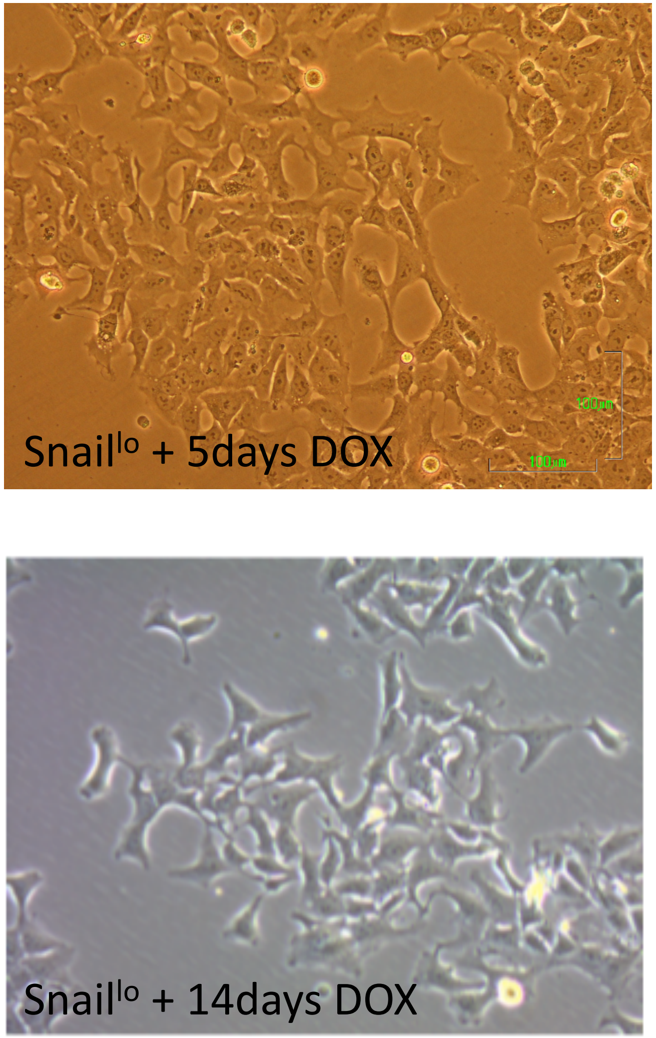

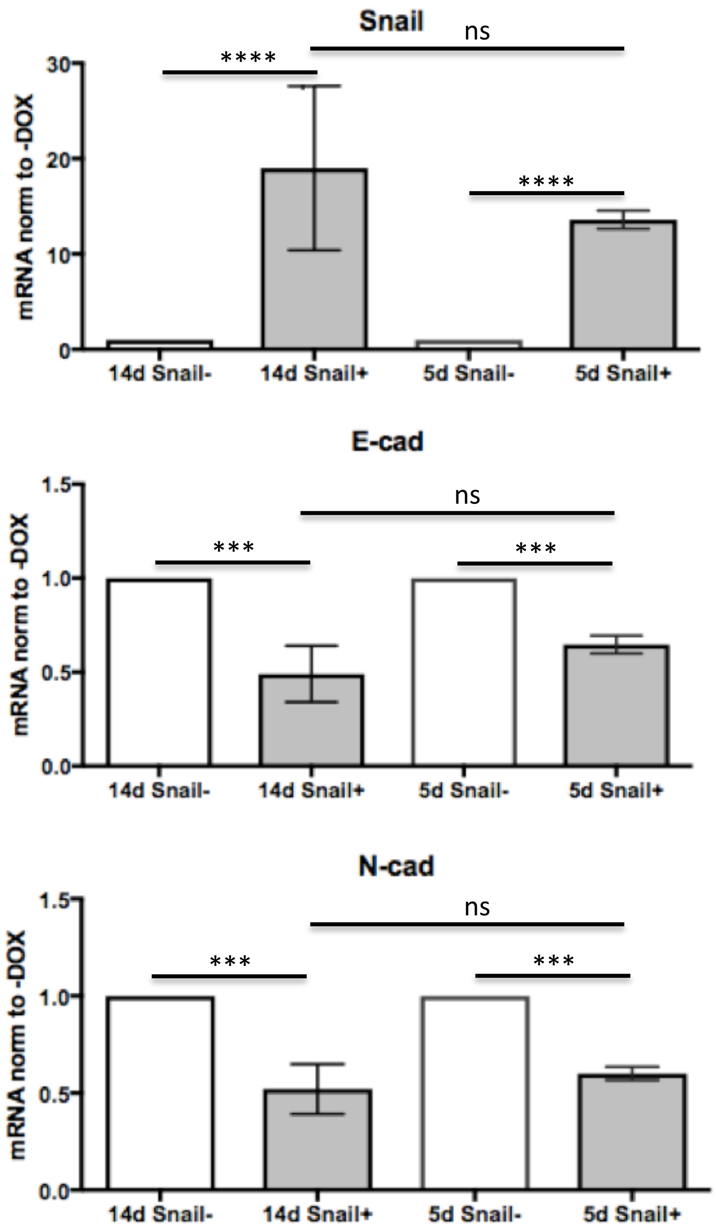

Supplementary Figure 3 - Comparison of 5 and 14 days DOX incubation of Snail-lo

(A) Phase contrast images of Snail-lo cells incubated with DOX for 5 or 14 days. (B) Expression levels of genes shown at 5 and 14 days in the presence (+) and absence (-) of DOX.
