# Supplementary material for: Single-copy Snail upregulation causes partial epithelial-mesenchymal transition in colon cancer cells
Source: BMC Cancer. 2023 Feb 14;23:153. doi: 10.1186/s12885-023-10581-3 (PMC9926732; doi:10.1186/s12885-023-10581-3)
Supplement: Supplementary file 4 — Additional file 4. [file 12885_2023_10581_MOESM4_ESM.pdf]

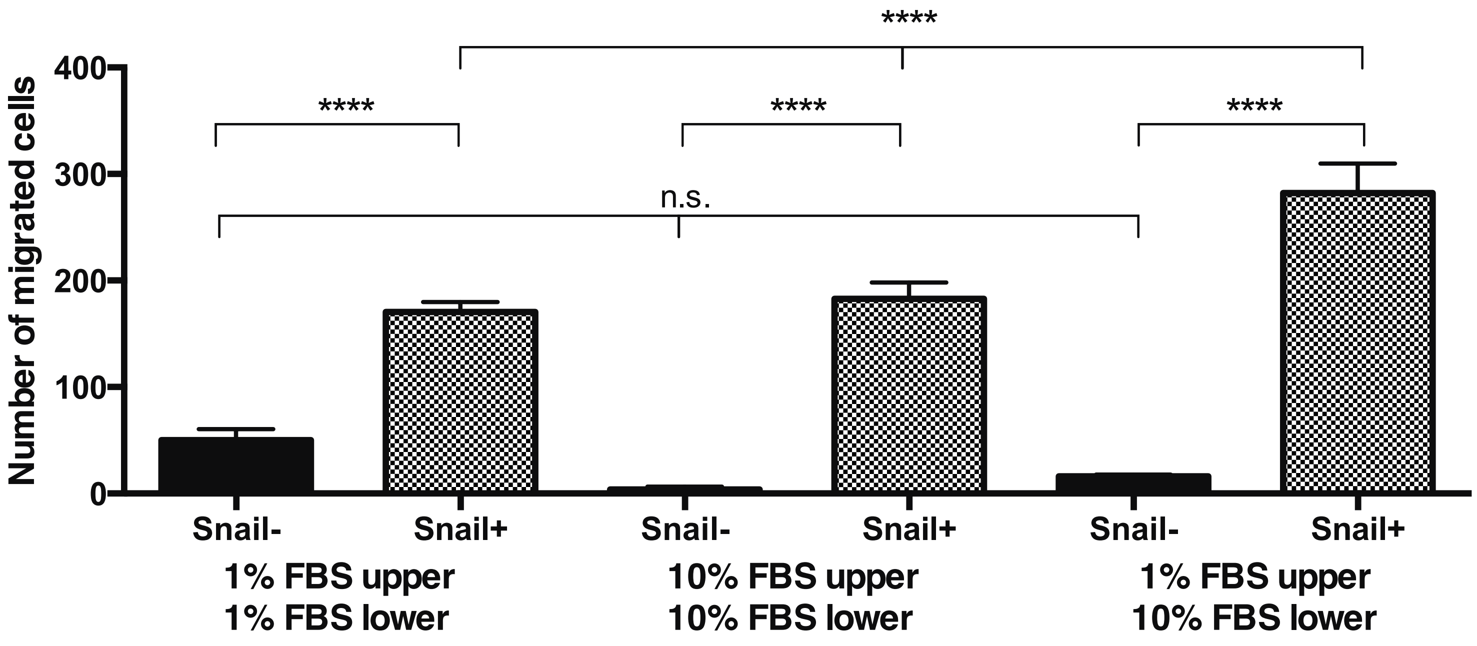

Supplementary Figure 4 - Snail expression causes increased migration in a Transwell assay.

Bars show the number of cells migrating through the membrane at 24 hours. Error bars = SEM. -/+DOX. n=3 biological replicates with 3 technical replicates each. One-way ANOVA. ns=not significant, ****p<0.0001.
