# Supplementary material for: Single-copy Snail upregulation causes partial epithelial-mesenchymal transition in colon cancer cells
Source: BMC Cancer. 2023 Feb 14;23:153. doi: 10.1186/s12885-023-10581-3 (PMC9926732; doi:10.1186/s12885-023-10581-3)

Whole blot 1A

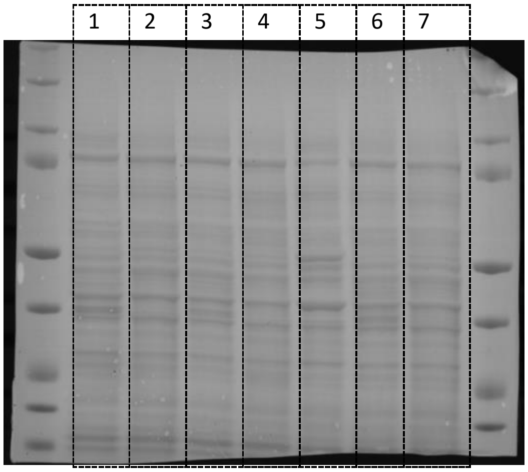

Lane	Sample
1	Empty episome + DMSO
2	Empty episome + DOX
3	SnailHi + DMSO
4	SnailHi + DOX
5	IMR90 (mesenchymal cell line)
6	Episome with AP4 (original construct from German lab) + DMSO
7	Episome with AP4 (original construct from German lab) + DOX

E-cadherin

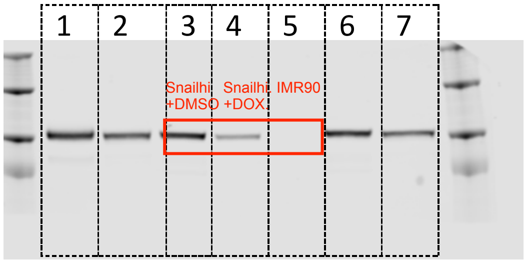

Fibronectin

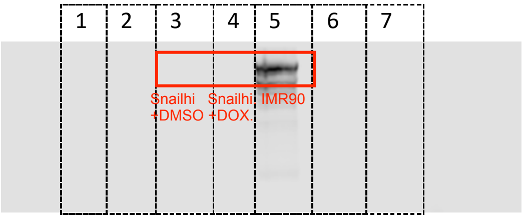

GAPDH

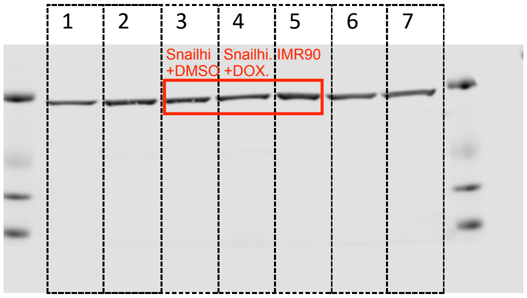

Whole blot 1B

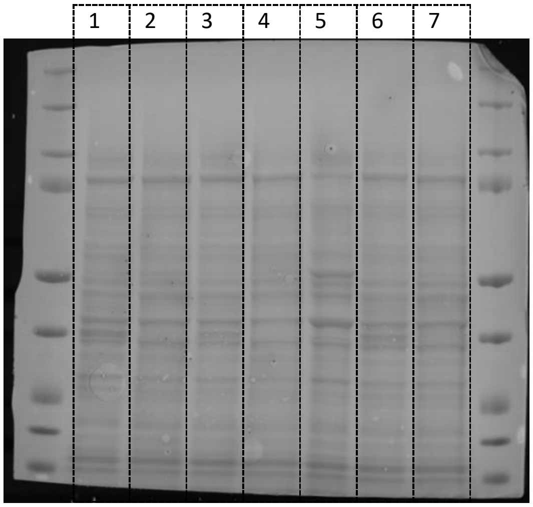

Lane	Sample
1	Empty episome + DMSO
2	Empty episome + DOX
3	SnailHi + DMSO
4	SnailHi + DOX
5	IMR90 (mesenchymal cell line)
6	Episome with AP4 (original construct from German lab) + DMSO
7	Episome with AP4 (original construct from German lab) + DOX

N-cadherin

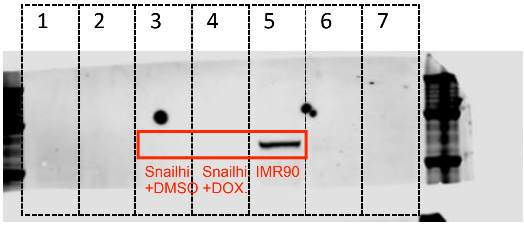

Snail

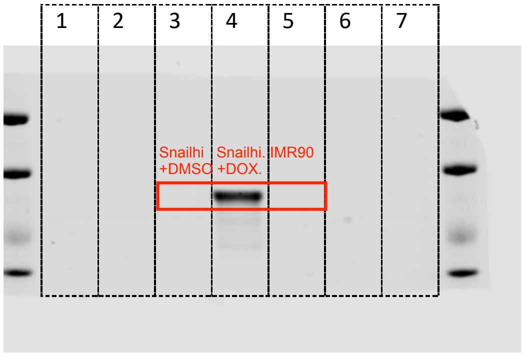

Vimentin

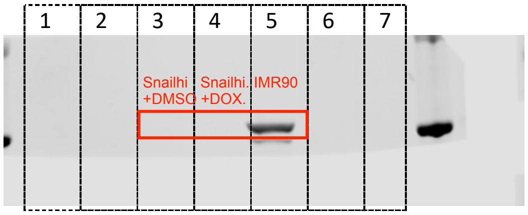

Whole blot 2A

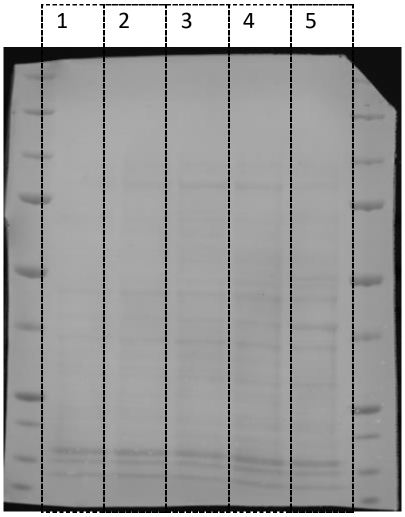

Lane	Sample
1	SnailLo +DMSO
2	SnailLo +DOX
3	Control (CreOnly inserted) + DMSO
4	Control (CreOnly inserted) + DOX
5	IMR90 (mesenchymal cell line)

E-cadherin (upper bands) and B-catenin (lower bands)

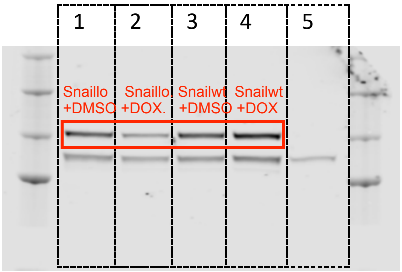

Fibronectin

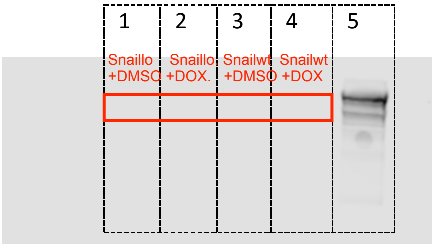

GAPDH

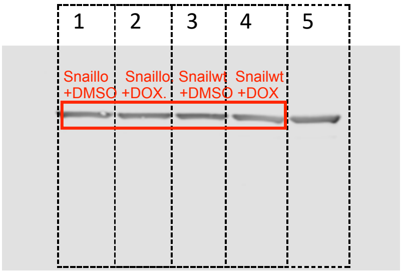

Whole blot 2B

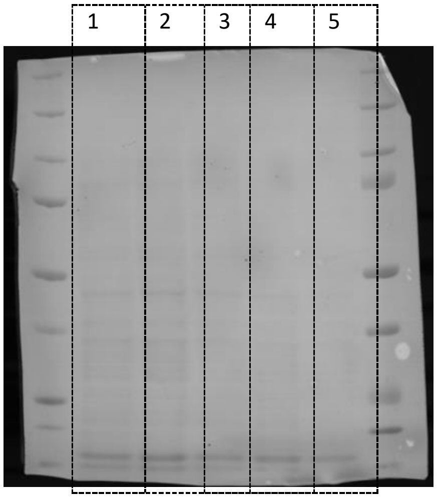

Lane	Sample
1	SnailLo +DMSO
2	SnailLo +DOX
3	Control (CreOnly inserted) + DMSO
4	Control (CreOnly inserted) + DOX
5	IMR90 (mesenchymal cell line)

Snail

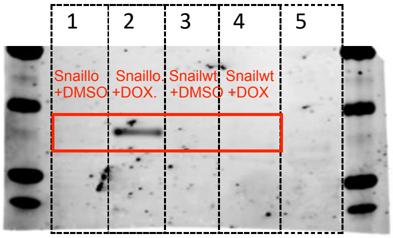

N-cadherin

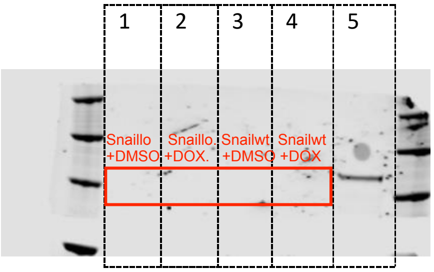

Vimentin

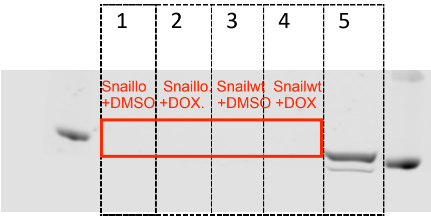

Supplement: Supplementary file 6 — Additional file 6. [file 12885_2023_10581_MOESM6_ESM.pdf]
